# Humeral Head Articular Cartilage Damage by a Retained Arthroscopic Suture Anchor Inserter in a College Athlete

**DOI:** 10.7759/cureus.44536

**Published:** 2023-09-01

**Authors:** Robert Wood, Christopher McCrum

**Affiliations:** 1 Orthopedics, Samaritan Health Services, Corvallis, USA; 2 Orthopedic Sports Medicine, Samaritan Health Services, Corvallis, USA

**Keywords:** retained surgical bodies, suture anchor, arthroscopic labral repair, orthopaedic arthroscopic surgery, arthroscopic bankart, arthroscopic complications, arthroscopic surgery, arthroscopic shoulder surgery

## Abstract

Arthroscopic repair of the glenoid labrum has been shown to be a safe and effective method to address recurrent shoulder instability. However, suture anchor devices must be used appropriately and with caution to avoid postoperative complications such as prominence, migration, and loosening, which can result in intraarticular pathology postoperatively. We report a case of significant glenohumeral articular cartilage damage that occurred as a result of a retained broken suture anchor inserter after arthroscopic Bankart repair. This case demonstrates an uncommon but serious complication that can occur during the surgical insertion of suture anchors when performing arthroscopic labral repairs. Although rare, retained foreign bodies must also be considered in the differential of a symptomatic postoperative patient. Thus, postoperative radiographs are also of extreme importance in evaluating a patient with persistent pain and crepitus after surgical glenoid labral repair.

## Introduction

Arthroscopic repair is the gold standard procedural intervention to address shoulder instability [1]. While it has been proven a safe surgical technique, there are risks that are present during arthroscopic procedures. Retained surgical bodies in the intraarticular space represent one such risk that can be potentially devastating, especially in a young otherwise healthy athlete [2]. We report a case of this specific complication and describe the important steps that can be taken in order to avoid the retention of surgical instrumentation within the intraarticular space. 

## Case presentation

An 18-year-old right-handed male collegiate athlete presented with complaints of left shoulder pain and instability for several months. The patient had previously undergone arthroscopic posterior labral repair at an outside facility approximately three months prior to presentation. The patient’s initial postoperative period was uneventful, and he had made good progress with physical therapy evidenced by both increased range of motion and improvements in strength throughout, especially with forward flexion and external rotation. However, six months postoperatively, he was continuing to experience posterior shoulder pain. 

Upon presentation, the patient exhibited a normal neurovascular exam of the left upper extremity. However, he had some limitations in active internal rotation of the left shoulder when compared to the contralateral extremity. Additionally, the patient had a palpable and audible click when taking the shoulder through a normal range of motion. His physical examination was also remarkable for a positive posterior apprehension test and positive jerk test on the left side. The remainder of the physical exam and special tests were negative.

**Figure 1:**
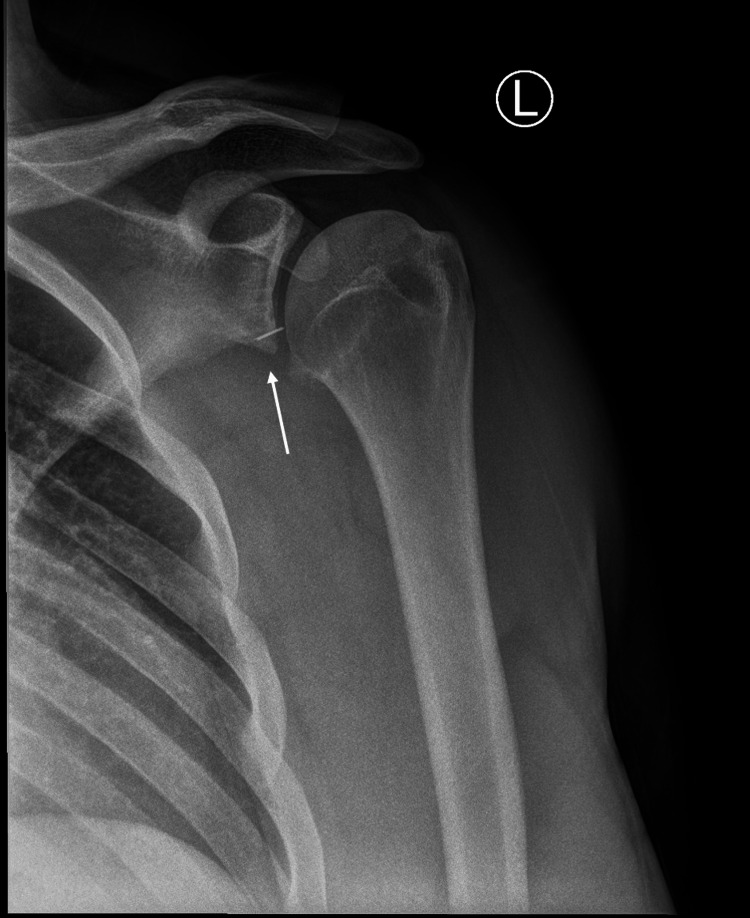
Grashey radiograph demonstrating retained metallic intraarticular body in the glenoid indicated by the arrow.

**Figure 2:**
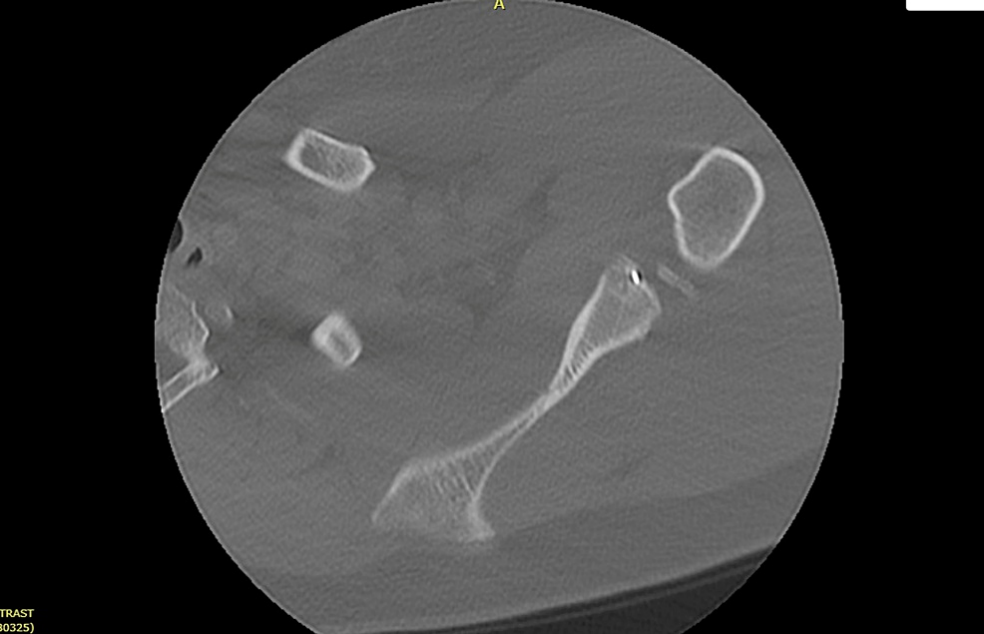
Axial cut CT image showing retained suture anchor at the anterior inferior glenoid rim.

Upon presentation, conventional radiographs were performed to include anterior to posterior (AP), Grashey, scapular Y, and axillary lateral views (Figure 1). Imaging was remarkable for the presence of a small metallic object most visible on the AP and Grashey views. The metallic object was not readily identifiable but appeared to be similar in shape to an arthroscopic anchor inserter. Given the presence of metal artifacts on plain film imaging, the decision was made to obtain a computed tomography (CT) scan of the left shoulder. The CT scan revealed the presence of tunnels consistent with the patient’s previous posterior glenoid labrum repair with a prominent metal object (Figure 2). The object appeared to be aimed into the anterior inferior tunnel but was not entering the tunnel. This was found to be slightly more prominent in the anterior inferior glenohumeral articulation. Additionally, there were some osteophytes present on the inferior aspect of the humeral head with some associated posterior joint space narrowing.

With these imaging findings, there was concern that significant cartilage loss had already begun to develop. Thus, the decision was made to perform left shoulder arthroscopy with implant removal and concomitant microfracture of the area of significant cartilage loss. Because the patient continued to have significant symptoms involving his left shoulder and had failed to progress with conservative management for nearly a year postoperatively, he elected to move forward with surgical intervention.

**Figure 3:**
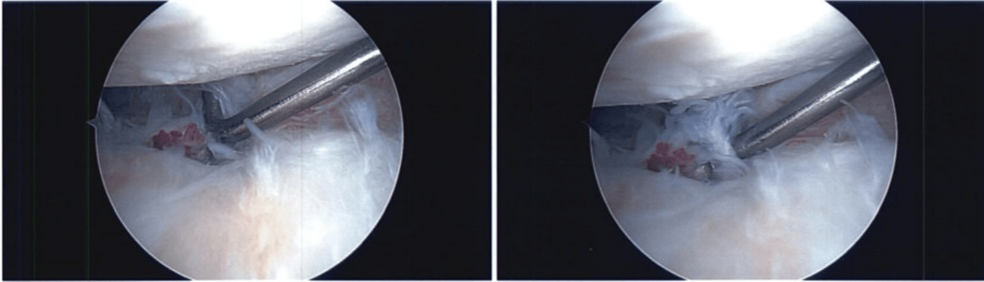
Arthroscopic view of the retained suture anchor inserter at the anterior inferior glenoid.

**Figure 4:**
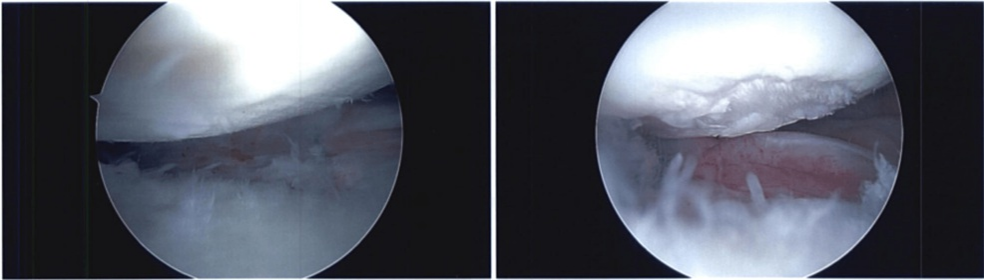
Arthroscopic view of the humeral head showing the area of denuded articular cartilage.

A diagnostic left shoulder arthroscopy was performed and showed mild fraying of the superior labrum with some mild tendinitis around the previously placed suture anchor. Upon examination of the anterior labrum, a prominent piece of metal was encountered protruding into the glenoid articular surface. It was found to be prominent by about 1-2 mm on the articular surface with a small amount of labral tissue draped over it (Figure 3). The humeral cartilage was found to have a 25 mm x 10 mm longitudinal area of denuded cartilage that correlated with the area where the prominent metal foreign body was located (Figure 4). The glenoid cartilage showed extensive degenerative changes throughout the entirety of the glenoid, worse in the posterior aspect of the glenoid. Additionally, there was grade III-IV chondromalacia extending nearly the entirety of the glenoid from the superior glenoid to about the 8:00 position.

After completing the diagnostic arthroscopy, a shaver and radiofrequency device were used to remove inflamed synovial tissue from the anterior, posterior, and inferior shoulder. A pituitary rongeur was used to remove the retained orthopedic device and a sub-centimeter loose body was excised using an arthroscopic shaver. Microfracture was performed on the chondral defect on the humeral head using an arthroscopic awl. Given the extensive changes on the glenoid side of the joint, no further surgical procedures were performed.

After surgery, the patient was placed into a sling for comfort and allowed to begin full active and passive range of motion with physical therapy at one week postoperatively. Strengthening was begun at six weeks postoperatively with abduction and external rotation strengthening deferred until the 12-week mark. The patient had no complications postoperatively and was able to regain the full range of motion of his shoulder with the resolution of all of his symptoms by the eight-week postoperative visit. Additionally, the patient was able to return to full participation in collegiate football at his previous collegiate position on the offensive line without incident.

## Discussion

Arthroscopic reconstruction is considered the gold standard for surgical treatment of labral tearing leading to shoulder instability. Notably, arthroscopic labrum repair has been shown to have excellent outcomes in competitive athletes with as many as 88% of patients returning to their preinjury level of participation [1]. However, there are several considerations that must be respected when performing arthroscopic repair of the labrum. Importantly, there are anatomic factors that must be taken into account. For example, the glenoid rim is narrow when compared to other areas where arthroscopic anchor insertion is commonly performed such as the humeral head [2]. The narrow nature of the glenoid rim has been shown to reliably accommodate relatively small anchors and can typically only tolerate anchors with diameters of 4.1 mm or less [3]. The anterior inferior glenoid labrum has been shown to have the least amount of available bone for anchor insertion and thus is frequently an area of difficulty in arthroscopic labrum repair. In this case, all of the inserted anchors appeared to be asymptomatic except for the anchor in the anteroinferior glenoid. This case again emphasizes the importance of focusing on the placement of this specific anchor as it can be difficult when compared to other areas of repair.

Retained surgical bodies are complications that can occur regardless of the type of surgical procedure performed. They occur more commonly in abdominal procedures and are most frequently sponges. The overall incidence of retained foreign bodies after surgery is estimated to be about one out of every 5,500 surgical operations [4]. Although this occurs much less frequently in upper extremity surgery, it is still a possible complication that can have devastating and lasting effects. This case demonstrates the importance of keeping retained surgical bodies as a differential diagnosis in the postoperative patient who presents with new onset symptoms not readily explained by normal postoperative changes. It underscores the importance of diligently investigating the possibility of a foreign object causing intraarticular symptoms with multiple imaging modalities and possibly addressing the issue surgically.

Complications after arthroscopic suture anchor repair in the shoulder have been extensively documented in the literature. However, to our knowledge, there have been no prior published case reports of retained suture anchor inserters. Zuckerman and Matsen have previously described glenohumeral complications associated with suture anchors and elaborated on four typical causes of the problems [5]. These were categorized as incorrect initial surgical placement, postoperative implant migration, implant loosening, and implant breakage. In the current case, implant failure was likely a component as a portion of the inserter appears to have broken off during the insertion of the arthroscopic anchor. 

Iatrogenic glenohumeral arthropathy is also a described complication after arthroscopic Bankart reconstruction [6]. Rhee et al. presented a case series of five patients who developed significant pain and crepitus with shoulder range of motion after undergoing arthroscopic Bankart reconstruction with metal suture anchors. In each of these cases, the cause was found to be protrusion of the anchor tip into the articular surface. All of these patients were found to have some associated chondral defects in the humeral head. Although the patient in the current case underwent posterior labral repair with bio-composite suture anchors, the resultant postoperative complication was similar due to the presence of metal foreign body protrusion into the glenohumeral articulation.

The present case also underscores the importance of obtaining appropriate postoperative imaging in patients who have undergone arthroscopic labral repair. This is especially true in patients that have symptoms concerning for suture anchor prominence or intraarticular foreign body including painful range of motion or new onset grinding and crepitus of the glenohumeral joint. The patient in this case underwent MRI arthrography in the postoperative period to evaluate the integrity of his labral repair. This was relatively unremarkable; however, metal does not create an MRI signal and is dark on MRI [7,8]. This likely explains the lack of significant findings on the postoperative MRI. Conversely, metal is radiopaque on conventional X-ray and thus may be the imaging modality of choice if there is any index of suspicion for retained metallic foreign body. Furthermore, CT scan can be a reliable adjunct imaging modality to improve the ability to adequately localize a foreign body when plain film X-rays are insufficient [9,10].

The patient presented in this case did extremely well postoperatively after undergoing arthroscopic foreign body removal. This emphasizes the importance of recognizing and removing any prominent hardware that appears to be contributing to a patient’s symptoms. This is especially true if the hardware appears to be damaging articular cartilage as it was in this case. Although a rongeur was able to be utilized in this case, many other arthroscopic techniques have been described including the use of osteochondral allograft harvesters and trephines [11]. With this in mind, any surgeon who performs surgical procedures involving the shoulder should be well-versed in techniques to remove prominent hardware both arthroscopically and open, if necessary, to address this kind of pathology.

## Conclusions

Arthroscopic Bankart repair is a generally safe procedure but does carry unique risks for complications. This case underscores the importance of maintaining a high level of clinical suspicion for potential complications in patients with continued pain and mechanical symptoms after the initial postoperative period. Clinicians should have a low threshold for ordering advanced imaging early in order to avoid potentially catastrophic damage to the humeral or glenoid cartilage, especially in young otherwise healthy athletes.
